# Supplementary material for: Are carbon emissions trading and green financial instruments synergistic? -Comprehensive quantitative research based on content analysis
Source: PLoS One. 2024 Mar 7;19(3):e0298601. doi: 10.1371/journal.pone.0298601 (PMC10919601; doi:10.1371/journal.pone.0298601)
Supplement: S1 Data — (ZIP) [file pone.0298601.s001.zip › Data/地级市Z_F工作报告.zip!/┴╔─■/┤≤┴1⁄4╩╨/└┤╫╘╣1⁄2╓┌║┼í╛┬φ┐╦╩2╛▌═°í┐_2013.docx]

各位代表：

　　现在，我代表大连市第十四届人民政府向大会报告工作，请予审议，并请市政协各位委员提出意见。

　　一、过去五年工作回顾

　　过去的五年，是极不平凡的五年。在党中央、国务院，省委、省政府和市委的正确领导下，市政府团结带领全市人民紧紧抓住东北老工业基地振兴和辽宁沿海经济带开发开放重大历史机遇，积极应对国际金融危机等严峻挑战，经受住了前所未有的困难和风险考验，圆满完成了十四届人大历次会议确定的各项任务，开创了大连科学发展的新局面。

　　五年来，全市地区生产总值年均增长14.2%;2012年达到7000亿元，是2007年总量的2.2倍；人均超过1.55万美元。公共财政收入年均增长22.9%;2012年完成750.1亿元，是2007年的2.8倍。固定资产投资年均增长24.8%;2012年按新口径统计完成5690亿元，是2007年的3倍。社会消费品零售总额年均增长17.8%;2012年完成2230亿元，是2007年的2.3倍。万元GDP综合能耗累计降低20%,主要污染物年均减排4.8%.城市居民人均可支配收入年均增长12.7%;2012年达到27480元，是2007年的1.8倍；农村居民人均纯收入年均增长14.1%;2012年达到16200元，是2007年的1.9倍。主要经济指标增速持续超过东部地区平均水平，保持了区域竞争中的优势地位。

　　（一）全域城市化加快推进，发展动力显着增强

　　发展空间全域拓展。多中心规划，组团式布局，链条式发展，不断优化全域空间布局和功能区格局。编制完成《大连市城市总体规划》、《大连市土地利用总体规划》，出台了加快推进全域城市化的若干意见、实施方案和相关政策。规划了主城区、新市区、渤海区域、黄海区域“四大组团”,城市空间沿渤、黄两海及沈大、丹大两线“V”字形不断延伸和扩展。实施新市区管理体制改革，整合成立了金州新区、普湾新区，大幅拓展了高新区、保税区、长兴岛临港工业区、花园口经济区发展空间，形成了一批布局合理、各具特色的经济区，辽宁沿海经济带重点园区达到19个，构建起多点支撑、全域开发开放的新格局。

　　发展能量充分释放。主城区以服务业为主的产业结构基本形成，钻石海湾、生态科技创新城、高新区“一带多园”、旅顺绿色经济区建设加快推进。新市区城市化、工业化水平不断提升，普湾新区“十大园区”初步形成，基础设施建设全面推进；金州新区“十大产业园区”载体作用突出，集聚功能进一步增强；保税区汽车物流城产业架构基本形成。渤、黄海组团迅速崛起，长兴岛世界级石化产业园区、花园口新材料和生物产业基地、瓦房店风电产业基地、庄河国家生态工业示范园建设加快。2012年，新市区与渤、黄海组团完成固定资产投资占全市62%,县域地区生产总值、财政收入、固定资产投资增幅分别高于全市2、13和3.7个百分点，为全市保增长作出了重要贡献。城乡居民收入比由1.8∶1缩小到1.7∶1,大连成为副省级城市中城乡收入差距最小的城市，北三市在全国百强县排名快步前移。全域城市化成为拉动大连跨越发展最强有力的引擎。

　　（二）“三个中心”建设全面展开，城市功能不断提升

　　国际航运中心主体框架已经形成。 “一岛三湾”核心港区建设加快，2012年全市港口集装箱吞吐量突破800万标箱，增幅位居沿海规模以上港口首位。启动太平湾临港经济区建设，加快推进港城一体化。长兴岛疏港高速公路等陆续建成，集疏运体系逐步完善。机场三期扩建工程竣工，新机场填海工程及沿岸商务区建设顺利推进，大连航空公司、汉华航空公司正式运营。大连机场开通欧美直达航线，2012年旅客吞吐量1333万人次，国际旅客吞吐量居全国第四。

　　国际物流中心功能不断完善。大窑湾保税港区功能向东北腹地延伸，建设大中型内陆港15个，开通集装箱铁路班列7条。建设了一批各具特色的物流、分拨和配送中心，培育引进物流及相关企业3500余家。煤炭、粮食等国际性交易中心正式运营，大连成为国家现代物流示范城市。

　　区域性金融中心地位基本确立。各项存款余额超过万亿元。引进和新设各类金融及融资机构315家，星海湾金融城等金融功能区建设加快。新设百年人寿等5家金融总部，大连银行开设8家异地分行，东北亚现货商品交易所等15家交易场所规范运营。跨境贸易人民币结算额占全省60%,金融中心指数位列东北第一。

　　（三）产业结构调整成效显著，经济转型步伐加快

　　支柱产业优化升级。瓦轴、大机床、中国一重、重工·起重、冰山等企业完成新一轮改造，装备水平和产品技术水平大幅提升。奇瑞汽车、中远造船、STX、英特尔芯片、恒力石化、一汽大众发动机、德国大众变速箱、远东工具等一批重大项目相继投产，东风日产、华晨特种车基地等项目建设加快，金州新区获批“中国汽车零部件制造基地”.石化、造船、装备制造、电子信息四大优势产业增加值增长1.7倍。超百亿企业由9户增加到13户，大石化销售收入超千亿元，一批千亿级产业集群形成雏形。

　　战略性新兴产业快速发展。一汽节能与新能源客车、德豪光电等项目竣工投产，大连成为国家首批节能与新能源汽车示范推广城市。海洋石油钻井装备、大功率电力机车等产品的研制及产业化取得重大突破。高新区软件和信息技术服务、大连湾装备、金州新区电子信息三个集群成为国家新型工业化产业示范基地。“辽宁舰”在大连建成入列。

　　自主创新能力显着提升。全市共有国家级研发机构24个，组建了新能源等10个产业技术创新联盟，取得高档数控系统等一批重要成果，获得国家级科技奖励42项，专利申请和授权量年均增长25%以上。大连科技创新园建设加快，旅顺农业科技园区进入国家级行列，瓦轴获评“国家技术创新示范企业”.全市高新技术产品增加值年均增长30%,我市成为国家创新型试点城市和知识产权示范城市。

　　现代服务业和城区经济取得长足进步。服务业聚集区建设加快，人民路CBD等服务业聚集区的规模和效应持续增强。中山区建成“中国商业名区”,西岗区成为辽宁沿海经济带首个全域性现代服务业聚集区。北方不夜港工程加快实施，温泉旅游不断聚集，乡村旅游持续火爆，服装节等会展节庆创新发展，成功举办国内首个国际性葡萄酒美食节。

　　都市型现代农业蓬勃发展。粮食产量连续10年实现大丰收。设施农业、精品农业等“八大农业”协调推进，获得“农产品地理标志登记产品”8个。加快建设海洋牧场，渔业经济运行质量和效益不断提高。国家级农业产业化龙头企业发展到20家，居副省级城市前列。创建都市型现代农业示范区68个。瓦房店市、庄河市被评为国家级现代农业示范区。

　　（四）基础设施建设力度空前，城乡环境品质明显优化

　　重大基础设施建设快速推进。城建累计投资2264亿元，是上一个五年的12.2倍。国际会议中心、大连北站、LNG接收站等项目竣工，哈大高铁顺利开通，城市地铁、南部跨海通道、渤海大道、红沿河核电、大伙房水库输水入连、三道沟净水厂改造等工程加快推进。建成土羊高速等7条高速公路和东联路、振连路等道桥工程，拓宽改造了中华路、西南路等，遍布全域的“四网一环”公路网络和“七纵七横”城市快速交通系统加快构建。我市成为“公交都市示范城市”.新建改扩建体育中心、人民文化俱乐部等文化体育场馆。

　　全面实施农村“三新”、“六化”工程。全部行政村及70%的自然屯实现通油路。扶持42个重点镇加强基础设施建设，小城镇综合承载能力全面提高。

　　公共服务设施不断加强。城市建设“五个一”和“三个一”工程实施项目415个。新建和改建公园38个，完成了滨海路木栈道等一批健身路径，东北地区首条城市绿道在金州新区正式启用。新建、维修排水管网51公里，新建、改造城区供水管网325公里，疏浚河道、加固堤防1300公里，水务现代化建设加快推进。

　　生态环境得到极大改善。近两年全社会造林绿化投入连续超过百亿元，全市林木绿化率提高到44.5%,城市绿化覆盖率提高到45.2%.国土和海洋综合管理水平不断提高。完成了大钢、大化、大水泥等企业搬迁。建成21座污水处理厂，城市污水处理率达到95%;全国首座污泥处理厂投入使用。中心城区生活垃圾焚烧发电厂投产运行。我市先后被评为国际花园城市、国家森林城市。

　　（五）改革开放不断深化，发展活力充分迸发

　　重点领域和关键环节改革迈出新步伐。政府性融资平台整改成效显著，37家公司完成整改。加快国有经济布局和结构调整，国企资产增长116.3%.大连港、重工·起重等实现整体上市。出台加快民营经济发展政策，民营经济增加值年均增长22%,占比提高到65.5%.农村改革全面推进。实施甘井子区综合配套改革，长兴岛成为省综合改革试验区。

　　对外开放成果丰硕。旅顺口区、长海县获准扩大对外开放。长兴岛临港工业区上升为国家级开发区，港口开放获得国家批准。实际使用外资连续三年超100亿美元，连续四年位居副省级城市首位。累计引进内资5706亿元。一批重大内、外资项目相继落户大连，其中世界500强和行业百强企业381家。完成了41个海外并购科技型中小企业项目。外贸出口年均增长11.5%.出口商品结构不断优化，出口市场扩展到全球196个国家和地区。成功承办两届夏季达沃斯论坛。

　　（六）社会事业繁荣发展，人民生活有效改善

　　教育事业持续快速发展。学前教育普及普惠实现新跨越，各地区义务教育均达到省定“双高普九”标准，5个区实现省基础教育强区目标，普通高中优质特色发展，大连职教基地高起点规划建设，高等教育“区校一体化”战略合作启动实施。

　　医药卫生体制改革取得新进展。国家基本药物制度惠及城乡600万市民，基本公共卫生服务均等化水平不断提高，医疗基础设施不断完善，新农合参合率达99%.投入3亿元资金，启动快速提升医疗软实力工程。健康家庭促进计划惠及百万家庭。

　　文化体育事业日益繁荣。“文化大连”等系列文化活动深入开展，市民文化生活日益丰富，文化体制改革基本完成。群众性体育活动蓬勃开展，竞技体育水平不断提高，“十二运”各项筹备工作顺利推进。

　　社会保障体系进一步健全。新增就业87.5万人，城镇登记失业率保持在3%以下，在全国“创业型城市”绩效评估中名列第一。实现了养老保障制度城乡全覆盖和基本医疗保险市级统筹，城乡居民和职工社会保险待遇逐步提高。社会救助体系建设成效显著，城乡低保对象和困难群众生活得到保障。住房保障体系加快构建，住房公积金覆盖面进一步扩大。投入6亿元资金，启动城子坦淹没区、华铜矿塌陷区及工矿棚户区整体搬迁。

　　社会管理创新取得实效。“无线中山”建设、西岗区365工作体系、沙河口区“帮万家”服务受到市民欢迎。建立信访联合接访机制，发挥仲裁和各类调解组织作用，有效防范和化解了社会矛盾。法治城市、平安大连建设扎实推进，3个城区建成“国际安全社区”.安全生产、应急管理体系进一步完善，应对公共突发事件能力进一步增强。食品药品监管不断强化。社会安全形势保持稳定。

　　财政支出结构不断优化，全市财政用于民生的投入达到1918亿元，年均递增23.3%.累计完成83件为民办实事项目。民族宗教、残疾人、外事、侨务、对台、人防、档案、防震减灾、民兵预备役等各项事业都取得长足进步。我市连续获得全国文明城市、全国双拥模范城荣誉称号。

　　（七）政府自身建设不断强化，服务水平进一步提高

　　政府机构改革顺利实施。进一步减少市政府工作部门，全面清理和规范了议事协调机构。

　　行政服务资源深度整合。市区两级行政服务体制统一联动，在国内率先建立了“五合一”集中行政服务体制。市区两级网上审批系统对接运行，非行政许可审批事项全面清理规范，政务公开全面推进。民心网、民意网和12345市民热线作用更加突出。行政机关导入质量管理体系工作全面铺开。

　　依法行政深入推进。制定法规草案、规章60多件，政府工作的制度化、规范化、法制化水平得到提升。坚持主动向人大报告工作、向政协通报重大事项，自觉接受人大法律监督、工作监督和政协民主监督、社会各界监督。认真办理人大、政协的建议、提案。落实重大行政决策社会听证、会议旁听、政府法律顾问等制度，政府行政决策更加科学民主。

　　勤政廉政不断加强。强化审计监督、行政监察，从源头上预防腐败，优化发展环境。在转变工作作风、提高行政效能方面取得显著成效，群众对政府的满意度不断提高。

　　各位代表，刚刚过去的2012年，是国际国内形势复杂多变、我市经济发展面临困难增多的一年。面对严峻挑战，全市上下团结奋进，共克时艰，努力保持经济社会平稳较快发展。

　　一是“稳增长”夺取新胜利。加强经济运行调度，多措并举帮助企业解决困难，安排2.5亿元资金支持企业技术改造，安排3亿元资金扶持外经贸发展，外贸出口逆势增长12%.大力拓展融资渠道，实现融资总额2500亿元。工业经济7月份先于全国止落企稳，全年规模以上工业总产值突破1万亿元大关，增加值增长11%.建筑业产值突破2000亿元。社会消费品零售总额增长16%,实际使用外资增长12%.预计实现地区生产总值7000亿元，增长11%;完成公共财政收入750.1亿元，增长15.2%;固定资产投资按传统口径计算超过8000亿元，增长25%.成功迈上“三个七”台阶。

　　二是民生保障再上新水平。市政府为民办16件实事全面完成。实现城镇就业21万人。新农保基础养老金由80元提高到100元。投入低保资金4.5亿元提高城乡低保和农村五保供养标准。新建90所社区养老服务中心，开办50所普惠性幼儿园。新农合最低筹资标准提高到320元。调研制定收入分配改革方案，推进解决区域间收入差距过大问题。体育新城各单体项目陆续竣工，卫生中心建设顺利推进。市民健身中心投入使用。顺利通过国家环保模范城市复核验收。城乡居民收入分别增长13.2%、14%.

　　三是经济发展方式转变迈出新步伐。加快各重点产业园区建设，科学规划和建设长兴岛石化产业园区，固特异环保搬迁、机车旅顺基地一期等项目竣工投产，为工业经济注入新活力。“两化融合”成效显著，物联网相关产业加快发展。大连再生资源交易市场开市。大力实施创造力产业发展示范工程，大工计控数控和机器人控制系统等达到国际先进水平，大连“设计城”顺利运营。全市规模以上工业单位增加值综合能耗同比降低4.5%,超额完成省政府下达的任务。

　　四是加强和创新社会管理实现新进展。“六五”普法、法治城市创建活动全面启动。严厉打击违法犯罪活动，全面完善立体化治安防控体系，有效化解各种不稳定因素。国家级危险化学品应急救援大连基地落成，大孤山化工园区管控一体化项目建设取得重要进展。社区基层自治能力得到加强，社会组织登记管理体制改革步伐加快。提高人口服务管理水平，流动人口动态监测取得实效。

　　五是软环境建设取得新成效。开展软环境建设年活动，各级政府部门围绕机关作风整治、提高行政效能等内容，扎实开展问题查摆、限期整改等工作，努力解决企业群众反映的突出问题，政府部门的服务水平明显提高。

　　各位代表，本届政府任期即将结束。回顾五年的历程，我们倍感欣慰！全市上下风雨同舟、众志成城，以更加坚实的步伐、令人瞩目的业绩，成就了一个辉煌的五年。

　　--这五年，是快步发展的五年。我们坚持抢抓机遇，把科学发展作为第一要务，一步一个脚印、一年一个台阶，经济总量连续突破“三个五”、“三个六”和“三个七”大关，为大连加快发展、率先全面振兴奠定了坚实基础。

　　--这五年，是开拓创新的五年。我们坚持解放思想，创造性地实施全域城市化战略，新市区面积拓展到2299.6平方公里，成为新的经济增长极；渤、黄海组团加快建设，产业基础和城市基础不断夯实。大连未来发展框架已经拉开，在新一轮发展中抢占了制高点。

　　--这五年，是惠民富民的五年。我们坚持以人为本，努力加强以改善民生为重点的社会建设，不断完善社会保障体系和公共服务体系，城乡居民收入分别增长81.9%、93.6%,人民群众得到更多实惠，大连正向全面小康社会加快迈进。

　　--这五年，是攻坚克难的五年。我们坚持依法行政，不断提高执政能力，凝心聚力，团结拼搏，有效应对国际金融危机严峻考验，妥善处置了一系列重大事故和事件。在经济社会深刻变革过程中，大连始终保持政通人和、和谐稳定的大好局面。

　　各位代表，大连振兴发展取得的重大成就，是党中央、国务院，省委、省政府和市委正确领导的结果，饱含着全市人民的辛勤汗水，凝聚着社会各界的智慧和力量。在此，我代表市政府，向给予政府工作大力支持的人大代表和政协委员，向各民主党派、工商联、人民团体和社会各界人士，向驻连部队和武警官兵，向老领导、老同志，向全市人民和关心支持大连建设发展的海内外朋友表示衷心的感谢！

　　我们应当清醒地看到，前进的道路上还面临不少困难和问题：经济快速增长与资源环境约束之间的矛盾日益突出，加快转变发展方式的任务更加艰巨；公共服务能力和社会管理水平与人民群众的期望仍有差距，居民收入、医疗、交通等民生工作仍存在薄弱环节；制约科学发展的深层次体制机制矛盾依然存在。对于这些问题，我们将高度重视并在今后工作中切实解决。

　　二、今后五年的目标任务和2013年工作建议

　　各位代表，党的十八大确立了全面建成小康社会的宏伟目标，为我市未来发展指明了方向。我们要站在新起点、瞄准新目标、实现新跨越，谱写大连科学发展新篇章。

　　今后五年政府工作的指导思想是：全面贯彻落实党的十八大精神，以邓小平理论、“三个代表”重要思想、科学发展观为指导，推进全域城市化、新型工业化、城市智慧化和农业现代化，加快“三个中心、一个聚集区”建设，提高经济增长的质量和效益，深化改革开放，改善民生质量，提前全面建成小康社会，为加快建设富庶美丽文明的现代化国际城市而努力奋斗！

　　今后五年我市经济社会发展的预期目标是：地区生产总值年均保持两位数增长，万元GDP综合能耗年均降低3.77%;主要污染物排放总量年均削减2%;公共财政收入、全体居民可支配收入实现倍增。

　　实现上述要求和目标，要着重把握好以下几点：

　　一是坚持全域城市化，推进城乡发展一体化。统筹城乡、区域和陆海发展，优化“四大组团” 的空间、产业和功能布局，依托辽宁沿海经济带19个重点园区，承接和引进高端产业，提升新市区开发建设水平，增强主城区辐射集聚功能，进一步夯实渤、黄海组团产业基础和城市基础，形成城市组团、中心城镇和小城镇合理配置的全域空间布局。着力在城乡规划、基础设施、公共服务等方面推进一体化，促进工业化与城市化良性互动，不断强化全域城市化的引擎作用。

　　二是坚持绿色低碳发展，着力构建现代产业发展新体系。抓住加快转变经济发展方式这一主线，把握扩大内需这一战略基点，立足发展实体经济这一坚实基础，以增量带动结构调整，以创新促进存量升级，推动工业化向纵深发展。大力培育战略性新兴产业，优化提升传统产业，加快发展现代服务业。不断提升新能源、新材料、海洋经济、都市型现代农业、旅游业等产业的贡献度。把生态文明理念和原则全面融入工业化、城市化全过程。

　　三是坚持创新驱动发展，切实增强城市发展动力。把科技创新摆在发展全局的核心位置，加快建设创新型城市。引导支持科技要素和创新资源向企业聚集，着力构建以企业为主体、市场为导向、产学研相结合的技术创新体系。充分发挥科研院所、重点实验室等各类创新平台作用，支持协同创新。大力推进创造力产业示范工程，助推优势产业转型升级。营造鼓励创新的社会环境，加大人才引进和培养力度。按照“政府主导、社会共识、市场运作”思路建设智慧城市，积极推进区域和行业试点。

　　四是坚持深化改革开放，充分激发城市发展活力。加大体制转型攻坚力度，完善有利于区域协调发展的体制机制。不断增强国有经济影响力，大力扶持非公有制经济发展。深化投融资体制改革，争取设立国家金融改革创新试验区和国际航运发展综合试验区。实施更加主动的开放战略，充分利用“两种资源”、积极开拓“两个市场”,着力培育开放型经济发展新优势，进一步强化大连在东北地区对外开放的龙头作用。

　　五是坚持着力改善民生，使人民群众充分共享改革发展成果。千方百计提高城乡居民收入，提前实现人均收入翻一番的目标。进一步完善就业促进、社会保障、社会救助、社会福利、住房保障体系，发展老龄服务事业和产业，健全覆盖城乡的公共服务和社会管理网络，推进基本公共服务均等化。认真倾听和解决群众普遍关注的热点难点问题，搞好重点民生工程建设，切实使改革发展成果惠及广大市民。

　　2013年是“十二五”承上启下的关键之年。全市经济社会发展的主要预期目标是：地区生产总值增长11%左右，万元GDP综合能耗下降3.77%.公共财政收入增长15%,固定资产投资增长20%,社会消费品零售总额增长16%,实际使用外资增长10%,全体居民可支配收入增长13%,城镇登记失业率控制在3%以下。

　　重点抓好以下八项工作：

　　（一）强化新市区示范带动作用，打造全域开发开放新优势

　　提高新市区建设水平。科学规划，突出重点，努力建设我国面向东北亚区域开放合作的战略高地、体制创新与自主创新的示范区、老工业基地转变发展方式的先行区、功能完善和现代宜居的生态区。重点推进作为新市区核心区的普湾新区建设，提升金州新区“十大产业园区”、保税区汽车物流城等功能水平。加快小窑湾、金渤海岸、金普城际铁路等基础设施建设，提升城市综合服务水平，带动产业和人口集聚，加快建设具有国际竞争力的先进制造业与现代服务业基地，打造引领东北地区全面振兴的重要增长极。

　　提升主城区辐射功能。进一步发挥中心城区对高端服务业的集聚功能，积极发展总部经济，培育城区经济新的增长点。推进大船重工搬迁，统一钻石海湾规划建设，加快大连金融服务区、西岗北部沿海商务区、星海湾金融商务区、西安路二期、甘井子区“两城四区”、高新区“一带多园”、旅顺口区“五城一都”开发建设，优先发展具有前瞻性的高新技术产业、绿色产业，引领全市产业结构升级。

　　加快渤、黄海经济区和城市带建设。重点推进长兴岛“十大园区”、瓦房店西郊工业园、庄河石化及化纤新材料园、花园口新材料和生物产业基地、长山群岛旅游避暑度假区建设，加快规划建设大连北黄海经济区。

　　实施“全域开放年”活动。积极推动利用外资向县域发展，努力构建以沿海重点园区为支撑的全域开放新格局。大力引进世界500强和行业百强企业，引导国内外企业在连设立研发中心和总部基地。引进内资增长20%.推进外贸转型示范基地建设，积极开拓新兴市场。加快“走出去”步伐，支持优势企业实施海外并购。办好第七届夏季达沃斯论坛。

　　（二）提高经济增长质量和效益，促进经济持续健康发展

　　狠抓工业经济运行。继续实施工业五项工程，完善工业经济运行预警监测体系，扶持实体经济发展，努力为企业排忧解难。继续加大投入，支持企业进行技术升级改造，提高产品竞争力。引导企业拓展新市场，提高产品市场占有率。推动营业税改增值税工作，减轻企业负担。

　　强化自主创新在转方式调结构中的核心作用。依托生态科技创新城、大连科技创新园和各重点实验室、企业技术中心打造技术创新平台，深化“区校一体化”建设，提高科技成果转化率。有效发明专利拥有量增长15%以上。组建网络化控制系统等10大产业技术创新联盟，新认定市级工程技术研究中心和重点实验室30家。

　　扶持中小企业发展。实施创新型中小企业培育工程，打造中小企业公共服务平台，改善小微企业发展环境，解决融资难、负税重等困难。民营经济各项经济指标增长15%以上。

　　加强生产要素保障。拓宽融资渠道，新增贷款和融资2400亿元。提高资源集约使用效率，土地、资金、电力等向重点项目、优势企业、新兴产业倾斜配置。实施“海外高层次人才引进工程”、“领军人才培养工程”和“专业技术人才知识更新工程”.办好“海创周”.

　　稳定投资拉动。进一步抓好重点项目建设，着力破解重大项目用地、融资难题，在要素资源配置上向重大项目倾斜。重大项目计划投资4000亿元。全面落实国家鼓励民间投资健康发展的新36条意见，引导支持民间资本进入公共服务领域和重点园区基础设施建设。

　　促进消费增长。努力提高居民收入，积极扩大消费需求。推进商业综合体、特色商业街和专业市场改造升级，深化万村千乡市场工程建设。完善社会信用体系，支持信贷消费、租赁消费发展。紧紧抓住哈大高铁开通机遇，扩大旅游、购物、会展等消费规模，促进房地产市场平稳健康发展。

　　（三）推进“三个中心、一个聚集区”建设，加快经济转型步伐

　　加快国际航运中心建设。推进港口结构调整和资源整合，全面开发太平湾临港经济区，加快长兴岛、旅顺、庄河组合港建设。加快船舶和船舶物资交易市场、航运人才市场建设，大力发展高端航运服务业，提升航运中心软实力。加快发展航空货运市场。壮大临港工业，活跃临港经济，提高本地货物比重，全力实现集装箱吞吐量超千万标箱目标。

　　加快国际物流中心建设。出台促进现代物流业发展的政策措施。加快大连水产品冷链物流园、香炉礁物流园、国际花卉交易中心建设。促进制造业与物流业联动发展，推动邮政快递行业安全、顺畅发展。培育本地重点物流企业10家，引进全球物流运营商2家。积极申办长兴岛综合保税区。

　　加快区域性金融中心建设。进一步强化服务功能，设立区域性股权交易市场，大力发展产业基金，创新发展科技金融、航运金融、绿色金融。扩大各金融功能区集聚效应，引进和新设各类金融及融资机构20家。支持大连商品交易所建设亚洲重要期货市场，推动“新三板”试点，继续推进企业境内外上市。

　　加快构建现代产业体系。促进传统产业优化升级，实现国金重工旅顺现代煤化工装备制造、大船重工长兴岛修船基地等在建项目投产，推进东风日产、长兴岛石化产业园区、中石油炼化一体化、一重石化装备基地等项目实施。加快推进高新区软件和信息技术服务、瓦房店轴承等产业集群建设。推动战略性新兴产业快速发展，培育发展数字家庭产业，推进“三网融合”试点，加快“无线城市”建设。优化海洋经济结构，推进长海海洋牧场、大连海洋经济产业园、旅顺海洋信息产业园等项目建设，实现海洋经济产值增长15%.加快推进服务业集聚区建设，加快发展总部和楼宇经济，大力培育研发设计、文化创意等高端服务业。

　　（四）加大统筹城乡力度，提高县域经济增长能力

　　增强县域经济活力。优化轴承、服装、家具、农产品加工等传统产业，积极培育装备制造、新材料等领域的重点项目和骨干企业。实施农村金融倍增计划，改善农村金融服务。加快涉农现代商品流通体系建设，新建农村商贸服务中心4个、超市20家。加快农村旅游资源开发，发展海岛休闲、温泉养生、农业节庆等旅游产业。加快县城和中心镇建设，培育一批工业重镇、旅游名镇、商贸强镇、都市型农业大镇。

　　加快都市型现代农业建设。全面提升渔业、畜牧、水果、蔬菜、花卉等优势产业发展水平，规划建设10个都市型现代农业园区，新发展设施农业10万亩，新植果树20万亩，创建水产健康养殖示范场20家。加强万亩粮食高产示范田建设，稳定粮食生产。推进农业标准化、产业化、规模化、集约化，大力发展农民专业合作示范社，培育农业龙头企业和名优产品。加强绿色生产，从源头上确保农产品质量安全。

　　落实强农惠农富农政策。完善村级公益事业建设一事一议制度，加快 “三新”工程建设，在200个村实施“六化”工程，创建10个新社区和50个新农庄。推进整村扶贫开发和生态移民搬迁，加快农业信息化工程，完善农业技术创新和推广服务体系。深化农村改革，健全土地承包经营权流转机制。切实保障农民利益，让广大农民共享城市化成果。

　　（五）加强城市规划建设与管理，加快建设美丽大连

　　严格执行城市规划。完成空港新区、体育新城和石灰石矿周边的控规和城市设计。做好重大基础设施项目、住宅建设用地、城市交通的规划安排，抓紧编制功能新区和中心城镇规划、海岸线资源保护与开发利用规划。

　　加强基础设施建设。全力推进新机场及沿岸商务区建设和长海机场扩建。加快城市地铁、渤海大道、长山大桥、机场快速路、丹大快速铁路等项目建设，建成中华路跨华北路、朱棋路立交桥，推进大连湾跨海交通、城际快轨延伸线等前期工程。改造供热管网200公里、供水管网50公里。继续实施城市建设“五个一”和县城建设“三个一”工程。全面推进国家水务现代化试点工作，完成大伙房水库输水入连、长海跨海引水工程，加快三道沟净水厂二期改扩建和“水上旅顺”项目建设，实施农村饮水安全工程。新建农村公路400公里。

　　推进生态市建设。严格企业能耗审计和限额管理，实现单位工业增加值综合能耗同比降低3%.加强公共机构节能管理。实施蓝天工程，加快拆炉并网和老旧住宅节能改造，建设农村综合整治示范项目。加强排污监管，主要污染物平均减排2%以上。实施马栏河下游环境综合整治工程，开工建设泉水污水处理二厂。加强矿山、建筑工地周边和重点区域环境整治。继续投入100亿元，建设青山生态系统工程。高标准完成体育中心周边和朱棋路两侧绿化建设工程。

　　开展 “城市环境提升年”活动。推动城市管理向做细、做精、做优转变，逐步建立科学化、精细化、常态化管理体系。

　　（六）实施重点民生工程，切实增进人民福祉

　　努力稳定和扩大城乡就业。发挥企业吸纳就业的主渠道作用，积极帮扶高校毕业生、失地失海人员和困难人群实现就业。优化全民创业环境，鼓励创业带动就业。加强职业技能培训，改善劳动力素质结构。全市实现城镇新增就业20.5万人，稳定就业率达到55%以上。

　　健全覆盖城乡的社会保障体系。完善统筹城乡的社会保障制度，逐步解决全域城市化进程中各类人员参加城镇职工养老保险问题。继续提高企业退休人员和城乡居民养老金标准。加快建立职工基本医疗保险门诊统筹制度，启动大连市工伤康复中心建设。探索建立居民医保重特大疾病保障机制。再次调整最低工资标准。深入开展劳动关系和谐企业、园区创建活动，依法维护劳动者合法权益。

　　完善基本民生保障体系。落实城乡低保标准自然增长机制和分类救助政策。加大特殊困难群体救助力度，完成1500户农村困难家庭危房改造。出台城乡社区养老服务中心运营管理办法，提升养老普惠化水平。推进市残疾人托养服务、脑康福利和军休疗养3个中心建设。支持慈善事业发展。

　　加快保障性住房体系建设。推进公租房配比建设工作，建立和完善公租房建设、运营、管理良性可持续发展机制，进一步加强住房公积金归集使用，研究出台困难群体公租房补贴政策。维修整治弃管房18万平方米以上。加快推进城子坦淹没区、华铜矿塌陷区及工矿棚户区整体搬迁。

　　加强市场监管和价格调控。完善政府储备和商业储备体系，抓好“菜篮子”工程，建设一级农副产品批发市场，继续办好社区便民店、蔬菜直销店和双休日市场，保持物价稳定。

　　推进“绿色交通”建设。继续实施城市交通综合整治，完善城市交通信息和城市慢行交通系统，疏通交通毛细血管，新建停车泊位2万个以上。优先发展城市公共交通，推进公交都市战略，新开、延伸、延时8条公交线路，更新公交客车731台，城市公交分担率达到45%以上。

　　（七）大力发展社会事业，提升社会管理水平

　　坚持教育优先发展。加强普惠性幼儿园建设。深入实施区际组团办学，推进义务教育均衡发展。实施农村中小学校车工程。提高普通高中公用经费标准，推动高中优质特色发展。加快职教基地建设，实行中职免费教育。支持在连高校内涵发展。

　　推进健康城市建设。继续实施快速提升医疗软实力工程。逐步开放医疗市场，增强全市整体医疗救治能力。新农合最低筹资标准提高到400元，基本公共卫生服务经费标准提高到每人每年35元。有序推进村卫生室实施国家基本药物制度。实现免费孕前优生健康检查城乡全覆盖。继续加强食品药品安全监管，确保群众饮食用药安全。

　　大力发展文化事业和文化产业。创建全国公共文化服务体系示范区，建设普湾新区大连博物馆、大连图书馆。注重文化遗产和老街、老建筑的保护与科学开发。全市所有公益性文化设施免费开放。实施文化产业振兴工程，推进大连文化产业创新园区、万达金石文化旅游等重大项目建设。

　　加快体育强市建设。全力做好“十二运”场馆建设、赛事组织、安全保卫等工作。以承办“十二运”为契机，完善城市功能，提升城市品质，凝聚城市精神。广泛开展全民健身运动，提高竞技体育水平，推动足球等运动迈上新台阶。

　　创新社会管理。加强法规、制度建设和基层服务，完善司法、仲裁和各类调解机制。大力推进信访工作体制机制创新，充分发挥各类社会组织作用，努力解决影响社会和谐稳定的突出问题。加强基层自治组织建设。完善社会治安动态防控体系，严密防范和打击违法犯罪活动。健全安全监管长效机制，开展隐患排查治理和重点行业领域安全专项整治活动。加强应急管控体系和联动机制建设，提高处置公共安全危机和突发事件的能力。出台质量发展规划，启动全国质量强市示范城市创建工作。搞好第三次全国经济普查。

　　继续做好民族宗教、残疾人、外事、侨务、对台、人防、档案、防震减灾、新闻出版、双拥共建、民兵预备役等工作。

　　（八）加强政府效能建设，提高公信力执行力

　　深入推进依法行政。认真做好人大、政协建议和提案办理工作，自觉接受人大法律监督、工作监督和政协民主监督以及社会公众、新闻舆论监督。着力建设法治政府，严格按照法定权限和程序行使权力、履行职责，进一步规范行政执法行为，着力提高行政机关工作人员特别是领导干部依法推动改革发展的能力。

　　切实提高行政效能。推进软环境建设常态化、制度化。进一步削减、调整行政许可事项，推进行政许可事项“应进必进”和向行政服务中心窗口“充分授权”;规范市、区两级非行政许可审批，建立非行政许可网上审批系统和电子监察系统。继续整合公共行政服务资源与功能，完善公共资源交易市场体系。深入推行政务公开和质量管理体系建设。

　　大力改进工作作风。践行改进工作作风、密切联系群众的有关规定，坚持深入实际、深入基层、深入群众，讲实话、干实事，敢作为、勇担当。精简会议活动、文件简报，切实改进会风、文风。

　　加强反腐倡廉建设。扎实推进惩治和预防腐败体系建设，加强财政资金监督和领导干部经济责任审计，努力从源头上防治腐败。加强绩效考核、效能监察和行政问责，切实纠正部门和行业的不正之风，着力解决损害群众利益、阻碍经济社会发展的突出问题。

　　各位代表！站在新的起点，展望美好前景，我们豪情满怀、信心百倍。让我们紧密地团结在以习近平同志为核心的党中央周围，在市委的坚强领导下，求真务实，奋发进取，顽强拼搏，扎实工作，加快建设富庶美丽文明的现代化国际城市，努力创造大连更加幸福美好的明天！
